# Historical Biography for Translational Medicine: An Important Genre for Translational Science

**DOI:** 10.3389/fcvm.2015.00009

**Published:** 2015-03-03

**Authors:** Simon W. Rabkin

**Affiliations:** ^1^Division of Cardiology, Department of Medicine, University of British Columbia, Vancouver, BC, Canada

**Keywords:** atherosclerosis, biography, coronary artery disease, peripheral vascular disease, translational medicine

Biographies are usually written about individuals who have fascinated us because of their accomplishments, opinions, or legacy. Although Western civilization recognizes the stories of the lives of biblical persons as well Greek and Roman philosophers and leaders, English literature attributes the first English biography to that of Samuel Johnson by James Boswell because of the detailed research which formulated the description of Samuel Johnson and the objectivity with which it was written. Historical biographies relate the biography to concurrent historical events and provide a historical background to the events of the person’s time. These kinds of biographies enlighten the reader on both the history of the time and its impact on the individual and when relevant to the role that the individual played in shaping the historical events. Historical fiction is a genre, which creates a fictional story surrounding real historical events. Although this genre has been spawned, in part, by the desire to learn more about a person or a period of history, it can sometimes be challenging for the reader to separate the fiction from the “reality” of history. All of these genres are enjoyable reading or escapes to another time and place for the reader.

The recent paper by Barton et al. (1) perhaps warrants defining a new genre – historical biography for translational medicine. The criteria for this genre should encompass (i) an accurate and comprehensive chronology of the relevant events and dates of the biographee, (ii) a description of the medical science at the time and the scientific question(s) that was (were) addressed, (iii) the other scientists/physicians who advanced the field to that point in time, (iv) the nature of the advancement made by the biographee, (v) the nature of the scientific obstacles that had to be overcome, (vi) the manner or method by which the person overcame the obstacles, (v) the method or manner of the advance from the fundamental research to clinical medicine, and (vii) the methods by which the person overcame the road blocks to the widespread utilization of the methodology and/or its utilization in the community or population at large.

Barton and co-authors have masterfully accomplished this task in their biography of Andreas Gruntzig. While there have been other biographies of this pioneer in the field of angioplasty, the biography by Barton and co-authors not only provides new details about their subject, obtained from newly identified documents and personal accounts from co-workers, but also provides considerable more insights into the character of the man and the challenges that he faced. This article accomplishes all of the above criteria for a historical biography for translational medicine.

Translational medicine or translational science in the field of medicine has attracted considerable recent attention. It grew from the concerns that the explosion of biomedical basic science was not being translated into meaningful improvements in patient care or not being accomplished in an expeditious manner commensurate with the large expenditures in research and the growing needs to treat patients or prevent disease occurrence (2). The problems facing the translation of medical science from basic research to widespread clinical practice have been described and discussed (2, 3). Obstacles for translational research have been dichotomized into two roadblocks. The first roadblock (labeled T1) refers to those obstacles impeding the “transfer of new understandings of disease mechanisms gained in the laboratory into the development of new methods for diagnosis, therapy, and prevention, and their first testing in humans” (2). A second roadblock (T2) occurs at the level of “the translation of results from clinical studies into everyday clinical practice and health decision making.” (2). Each of these roadblocks can have different origins and set of characteristics. It is likely that there are a number of important basic research accomplishments that have not been translated into clinical medicine because of T1 blocks. Similarly, there are likely useful procedures that have not overcome T2 blocks. Thus, there is a need to learn from people such as Andreas Gruntzig who successfully overcome these roadblocks.

In some curriculum, medical students are required to read a biography of a major medical figure. This exercise is designed to commemorate medical pioneers and provide a history of the biomedical tradition as part of a medical education. Historical biography for translational medicine can provide medical students and senior faculty with an understanding of how to overcome roadblocks in translational medicine.

To some extent, the accomplishments of a great person cannot be replicated by others without such talent. The characteristics, however, are still instructive and provide us with guideposts for career learning. What have we learned about Andreas Gruntzig from this biography? Success was accomplished by dedication, persistence, hard work, passion, unwavering dedication to attain the goal, and not being defeated by rejection. His comment that “*I have dedicated my life to vascular disease*” (1) stands in contrast to the often banal comment one might hear from many persons on their choice of career. The intensity of the “inner fire” that drove Andreas Gruntzig was evident to those around him (1). The intensity of his passion, however, did not prevent him from making friends in the field, some of whom were instrumental in overcoming the roadblocks.

Andreas Gruntzig was well trained in medicine and research as well as being exposed to leading medical “thinkers” and teachers in his training. This training included areas that superficially did not appear to be directly related to angioplasty such as public health and epidemiology but likely shaped his mode of thinking and problem solving. His education played a role in creating or nurturing the characteristics that are necessary for translational medicine. Hard work without any early recognition characterized his early years; “without any support from the federal funding agencies, working at home in the evenings and on weekends in addition to his full-time clinical duties, Grüntzig had succeeded in having functional, hand-made balloon catheters at his disposal” (1).

Medical administrative leadership, at its best fosters, creativity, and good biomedical science. At its worst, medical administration can throttle or extinguish great ideas and projects. Several times Andreas Gruntzig was discouraged from or told that he would not be able to do coronary angioplasty at his institutions. His persistence undoubtedly did not win him favor with his superiors or at his home institutions. Persistence in seeking out like minded individuals, however, was necessary for him to develop his concepts and techniques.

Barton and colleagues document through meticulous research, the many physicians and scientists who advanced the field to that point from where Andreas Guntzig made his leap forward (1). This kind of information pays tribute to individuals whose major contributions were the essential foundation of the field. It also recalls the medieval comment, later made famous by Sir Isaac Newton that one sees further standing “on the shoulder of giants” (4). In addition, the advancement of coronary angioplasty required a number of individuals who recognized the value of the work being done by Andreas Gruntzig and permitted him to proceed with his plans without necessarily directly contributing to the intellectual property of coronary angioplasty. Barton and colleagues document, the contribution of these facilitators and colleagues as well.

Andreas Gruntzig overcame the road blocks to the widespread utilization of coronary angioplasty by developing and leading teaching sessions and seminars on his techniques. These sessions drew people’s attention and kindled the “inner fire” in others to spread the technology to different medical centers.

Historical biographies for translational medicine need to be written by experts in the field together with those who know the details of the life and times of the biographee. It requires detailed research and the capacity to explain the biomedical issues, the process involved and the technical details of the subject matter. The value of the excellent contribution by Barton et al. (1) not only provides us with medical history and commemorates the advance but also provides us with important lessons to overcome some of the contemporary challenges in translational medicine.

## Conflict of Interest Statement

The author declares that he has no commercial or financial relationships or other interests related to the work presented that could be construed as potential competing interests.
